# Evaluating Toxic Interactions of Polystyrene Microplastics with Hazardous and Noxious Substances Using the Early Life Stages of the Marine Bivalve *Crassostrea gigas*

**DOI:** 10.3390/nano15050349

**Published:** 2025-02-24

**Authors:** Hoon Choi, Un-Ki Hwang, Moonjin Lee, Youn-Jung Kim, Taejun Han

**Affiliations:** 1 Maritime Safety and Environmental Research Division, Korea Research Institute of Ships and Ocean Engineering (KRISO), Daejeon 34103, Republic of Korea; moonjin.lee@kriso.re.kr; 2 Tidal Flat Research Institute, Marine Environment Research Division, National Institute of Fisheries Science (NIFS), Busan 46083, Republic of Korea; vngi1@korea.kr; 3 Department of Marine Science, Incheon National University, 119, Academy-ro, Yeonsu-gu, Incheon 22012, Republic of Korea; duckyj@incheon.ac.kr; 4 Department of Animal Sciences and Aquatic Ecology, Ghent University, Westenschapspark 1, Bluebridge, 8400 Oostende, Belgium; taejun.han@ghent.ac.kr

**Keywords:** polystyrene microplastic, microplastic toxicity, Pacific oyster, toxic interaction, cadmium, phenanthrene

## Abstract

Plastics pose a significant threat to marine ecosystems, owing to their slow biodegradability. Microplastics (MPs), in particular, affect marine life and maricultural organisms and can enter the food chain via ingestion by marine organisms, leading to bioaccumulation in predators, including humans. This study assessed the toxic interactions between polystyrene microplastic particles (PSMPs) and cadmium (Cd) and phenanthrene (Phe) using marine bivalves. While PSMPs were non-toxic to Pacific oysters (*Crassostrea gigas*), the toxicity of Cd and Phe was concentration-dependent. In most conditions, PSMPs reduced the toxicity of Cd and Phe, but in simultaneous exposure, they acted as Cd messengers, altering the toxicity during the adult stage. This study confirms that PSMPs can interact with coastal environmental pollutants, thereby accelerating biotoxicity and posing a significant threat to marine wildlife, mariculture, and human health. It also highlights the need to assess MP toxicity in coastal environments and their interactions with pollutants.

## 1. Introduction

We live in a period of history termed the Plastic Age [1,2]. Plastic is characteristic of the Anthropocene [3,4,5]. Global plastic production was estimated to be 367 million tons in 2020 [6]. It is projected to increase to 33 billion tons by 2050, with the COVID-19 pandemic accelerating its production rate [7,8]. Owing to high production and improper disposal, a considerable amount of plastic has been introduced into the environment. Approximately 10% of plastic waste produced is found in the marine environment [9]. Moreover, the predicted plastic waste discharged into marine ecosystems is estimated to reach 100–250 MT by 2025 [10,11,12,13,14].

Primary plastic waste produced by industrial activities is discharged into the marine environment and forms secondary plastic fragments through ultraviolet degradation and physical collision [10,11,15]. Manufactured or decomposed plastics in the size range of 1–1000 μm are called microplastics (MPs) [16]. Discharged MPs accumulate in the environment and affect marine ecosystems, marine life, and maricultural organisms, owing to their high durability and slow biodegradability [13,17,18,19,20]. Microplastics attach to the gut and gills of marine organisms, lowering their energy efficiency and reducing reproductive growth and muscle function [21,22,23,24]. Therefore, MPs in maricultural organisms are a cause for concern, owing to their potential impact on food safety [25].

Polystyrene (PS), the primary MP pollutant, is used in various products, such as food packaging (dairy and fishery), building insulation, electrical equipment, and eyeglass frames, because of its stable chemical properties, high strength, easy regeneration, good swellability, and low cost [25,26,27]. Polystyrene is a major pollutant in certain regions and is found in seawater, sediments, and organisms. For instance, PS was a predominant sediment-associated contaminant in 3 lakes in Italy [28] and 24 waterbodies, including lakes and urban estuaries, in China [29]. Furthermore, discharged PS remains afloat in seawater, owing to its low density (0.96–1.05 g/cm^3^). Thus, marine organisms such as bivalves could easily transport PS from the seawater surface to the intertidal zone [27,30]. Notably, PS is a major MP reported in bivalves, such as oysters and mussels, available in the Korean market [31].

Owing to their small size, MPs can enter the food chain via ingestion by marine organisms and subsequent bioaccumulation in predators [32]. Thus, MPs impact various organisms in or near the sea, such as mollusks, crustaceans, fish, turtles, mammals, and birds [33]. The ingestion or accumulation of microplastics may induce various physical and chemical toxic effects, including mechanical injury [34,35,36,37]. Additionally, owing to their structure, they act as carriers for toxicants, including chemical additives, such as plasticizers (e.g., phthalates and bisphenol A), colorants, and flame retardants, during the manufacturing process. They can also adsorb other organic contaminants from the surrounding marine environment [38,39,40,41,42]. Therefore, MPs, along with other coastal environmental contaminants, pose a threat to the marine organisms [43,44,45] and humans who consume these organisms.

Cadmium (Cd), a highly toxic trace metal and one of the most abundant environmental contaminants, is significantly elevated in rivers, estuaries, and coastal waters [46,47,48]. Oysters are known to accumulate Cd in their bodies at levels up to 104 times greater than that of seawater, exhibiting the highest bioconcentration factor (BCF) among bivalves and surpassing the BCF of other heavy metals [49,50]. Therefore, Cd poses a substantial threat to oysters and is recognized as an organic pollutant that can be absorbed by microplastics.

Phenanthrene (Phe), a member of the polycyclic aromatic hydrocarbons (PAHs), is classified as a persistent organic pollutant (POP) under the Stockholm Convention established in May 2001 [51] and is categorized as a Group 1 carcinogen by the International Agency for Research on Cancer (IARC) [52,53,54]. Once released into the environment, PAHs can be transported to the ocean either directly or indirectly [55,56,57,58,59,60,61,62]. However, PAHs are characterized by low water solubility and resistance to degradation, leading to their persistence and accumulation in the organic matter of coastal and benthic environments, which can harm these ecosystems [63,64]. Notably, Phe is found in higher concentrations than other PAHs in marine environments [54,64,65]. The presence of Phe in marine ecosystems is of great concern for human health, as it bioaccumulates through aquatic organisms and increases in concentration within seawater and sediments.

While there have been studies exploring the toxicity of various microplastics, a debate persists regarding the combined toxicity of microplastics simulating environmental concentrations and known pollutants in coastal environments.

Therefore, to assess the extent of microplastic bioaccumulation and its interaction with other toxic pollutants, we studied the marine invertebrate *Crassostrea gigas*, the Pacific oyster. We investigated the toxic interaction between PS microplastic particles (PSMPs) and the representative pollutants of the coastal environment, Cd and Phe, to understand the environmental risk of combined toxicity of marine pollutants. Additionally, we assessed the changes in toxicity values when the animals were exposed to MPs in conjunction with hazardous and noxious substances. Therefore, the potential effect of MPs in the environment was assessed for their combined toxicity with Cd and Phe. Furthermore, this study makes a novel contribution to the literature by confirming that MPs can either increase or decrease the toxicity of harmful substances.

## 2. Materials and Methods

### 2.1. Organism Preparation

We collected Pacific oysters, *C. gigas,* the test organism used in this study, from Iwon-myeon, Taean-gun, Chuncheongnam-do, Korea (36.5462° N, 127.6195° E), in August 2020, which coincided with their reproductive phase. They are most suited for toxicity analysis during this phase. After harvesting, the *C. gigas* were immediately placed in pre-chilled ice containers and transported to the laboratory. According to Kim and Chin [66], invertebrates similar to *C. gigas* can potentially maintain their physiological activities, such as oxygen consumption and water filtration rates, under optimal growth conditions. In this study, cleaned *C. gigas* were maintained in a 1-ton running-filtered natural seawater aquarium for one week at 20 °C and a 16:8 light:dark period. The physiological activity of the experimental organisms was averaged, and physically similar test organisms were used by measuring their size and weight.

### 2.2. Toxic Solution with PSMP Preparation

A preliminary range experiment was conducted to establish the test concentration of the coastal environmental pollutants, Cd and Phe, used in this study. Dimethyl sulfoxide (DMSO, Sigma-Aldrich, Inc., St. Louis, MO, USA) was used as a carrier solvent for preparing high concentrations of toxic stock solutions, and the actual exposure concentration of DMSO did not exceed 0.01% in the final toxic solution [67]. The toxic solution was serially diluted with natural seawater filtered using a 0.45 µm Whatman polyamide membrane filter (Cytiva, Incheon, Republic of Korea). The concentrations of the prepared Cd and Phe toxic solutions were 0–40 mg/L and 0–0.2 mg/L, respectively.

In general, MP concentrations in the marine environment are directly associated with the extent of pollution in that region. Additionally, the background concentration of the habitat affects the physiological activity of organisms. Therefore, confirming the actual environmental concentration of MPs or pollutants is essential before setting the test concentration.

Coastal and estuarine areas of the Korean southwest coast, the sample collection sites used in this study, face a significant threat posed by MP pollution, as their MP concentrations are the second and third highest in the world, respectively [68]. The average concentrations of MPs were 152 ± 92 particles/L and 211 ± 117 particles/L in Incheon-Gyunggi Bay [69] and Jinhae Bay [70], respectively, which were higher than those found in other countries.

Polystyrene fragments were pulverized using a stainless-steel grinder. Organic additives were removed by vertically shaking the pulverized PS fragments in a sufficient quantity of benzene using a vertical shaker (Jeiotech, Daejeon, Republic of Korea) [71,72]. Thereafter, the PS fragments were thoroughly dried at 50 °C in an oven, washed with distilled water, and stained using 0.1 mg/L Nile red solution (in 90% EtOH) to observe their size and shape [73,74]. Simultaneously, PS fragments were qualitatively analyzed using Fourier transform infrared spectroscopy. The final concentration of PS fragments with coastal environmental pollutants for the toxicity test was 300 particles/L.

However, when the PS fragments were exclusively considered for the toxicity test, the concentration was 0–1000 particles/L. Tween 80, used for homogenizing MPs, was below 0.0002% in the final solution [75,76]. In the final solution, PS fragments were separated by size as follows: <50 µm, 50–100 µm, 100–150 µm, 150–300 µm, 300–500 µm, 500–1000 µm, and >1000 µm, and their ratios were 63.4%, 13.3%, 8.9%, 7.6%, 5.5%, 1.2%, and 0.1%, respectively (Table S1).

### 2.3. Toxicity Test

Fresh sperm and egg germ cells from the *C. gigas* were selected for the germ cell and early life stage toxicity tests. First, the cleaned shells of fresh mature oysters were opened, and the gonads were scratched with a sterilized cutter. Sperm and eggs oozing from the lesions were collected for 30 min in a small beaker containing autoclaved seawater. The collected sperm cells and eggs were gently rinsed, and the microparticles were discharged. Thereafter, they were examined under a microscope to evaluate their conditions for germ cell toxicity and embryotoxicity tests (Table 1).

For the fertilization rate experiment, 10 mL of each concentration of the toxic solution was transferred to a 6-well plate (SPL life science, Pocheon, Republic of Korea), and 1 µL of sperm solution was added to each well. After exposing the sperm to toxicants for 30 min, 100 to 150 eggs were added to the test solution. During the exposure period, the eggs and sperm were incubated at 25 °C. The fertilization rate was calculated according to Equation (1).

(1) $$Fertilization\;rate\left(\%\right)=\left(\frac{Polocyte\;formed\;eggs}{Total\;counted\;eggs}\right)\times 100$$

**Figure 1:**
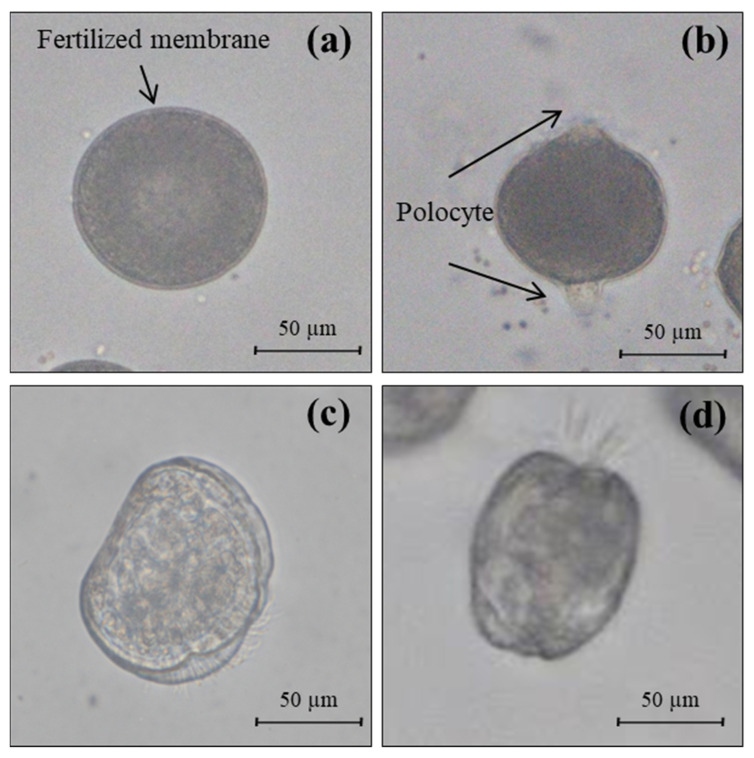
Developmental stages of *Crassostrea gigas*: (**a**) fertilized eggs with a fertilized membrane, (**b**) fertilized eggs with polocyte (developed polar bodies), (**c**) observable D-shaped embryo after 30 h, and (**d**) normal embryo with motility. When fertilization occurs normally, a fertilization membrane is formed around the zygote, and the polar body is released. In this process, the presence or absence of a developed polocyte and the development of a D-shaped embryo can be observed using a microscope (IX53, Olympus, Seoul, Republic of Korea).

The fertilization rate was determined by calculating the percentage of fertilization membrane and polocytes (a polar body formed in the eggs) to the total number of eggs (*n* = 100) through microscopic observation after 30 min of incubation (Figure 1a,b).

To estimate the D-shaped embryogenesis rate in *C. gigas*, 10–15 fertilized eggs were placed in different concentrations of toxic solutions (per mL) and observed after 30 h of exposure. Fertilized eggs were incubated at 25 °C, 100 µmol photons m^2^/s, and a 16:8 light:dark period. The culture medium remained unchanged during the experiments. The fertilized eggs transformed into normal D-shaped embryos during 30 h of exposure, classified by microscopic observation (Figure 1c,d). The D-shaped embryogenesis rate for the 100 embryos was calculated according to Equation (2) (*n* = 100).

(2) $$D\text{-}shaped\;embryogenesis\;rate\left(\%\right)=\left(\frac{Normal\;D\text{-}shaped\;embryo}{Total\;counted\;observed\;embryo}\right)\times 100$$

For the survival rate analysis, mature and fresh *C. gigas* were acclimated for 7 d prior to use. For the acclimation period, sufficient *Isochrysis galbana* (1–2 × 10^6^ cells·mL/L per one *C. gigas*) was supplied to the *C. gigas* once a day. Prior to the toxicity test, the physiological activity of the test species was averaged by using physically similar test organisms and measuring their size and weight. Six oysters, similar in size and weight, were placed in a 2 L beaker containing the solution for each tested concentration of the toxic solution. Each beaker was incubated at 25 °C and 100 µmol photons m^2^/s with a 16:8 light:dark period. The culture medium was not replaced during the experiments. The survival rate was calculated according to Equation (3).

(3) $$Survival\;rate\left(\%\right)=\left(\frac{Survived\;Oysters}{Total\;Oysters}\right)\times 100$$

The acclimated adult *C. gigas* samples were used for the survival rate analyses. The shell height, shell length, shell width, and wet weight of the *C. gigas* used in this test were averaged at (±CI; confidence interval) 19.62 mm (±0.96), 57.06 mm (±4.05), 31.18 mm (±2.14), and 5.53 g (±2.17), respectively.

### 2.4. Toxicity Value and Data Analysis

The toxicity values of Cd and Phe were analyzed using the probit analysis method to estimate the 50% effective concentration (EC_50_) and 95% confidence limit (95% Cl) of the fertilization rate, embryogenesis rate, and survival rate. Additionally, we used Dunnett’s test to analyze the non-observed effective concentration (NOEC) and lowest observed effective concentration (LOEC). The toxicity values were calculated using Toxcalc 5.0 (TidePool Scientific Software, McKinleyville, CA, USA, https://www.tidepool-scientific.com (accessed on 11 February 2025)).

Statistical analyses were performed using IBM SPSS statistics software (ver. 26; IBM Inc., Armonk, NY, USA). Significant differences between groups were identified using one-way analysis of variance (ANOVA). The significance level was set at *p* < 0.05.

## 3. Results and Discussion

### 3.1. Toxicity of PSMPs Toward C. gigas

The early life stages of *C. gigas* include sperm, egg, embryo, and larva. Their early embryo development is crucial and characterized by intense cellular activity. Therefore, any biochemical and physiological disturbance during early embryo development can cause malformations in the larvae [77,78]. The fertilization, D-shaped embryogenesis, and survival rates, which have been enacted and used for the Ecotoxicity Standard Test Method for 2018 in Korea, have been used to evaluate the toxicity of pollutants in various studies [79,80].

**Figure 2:**
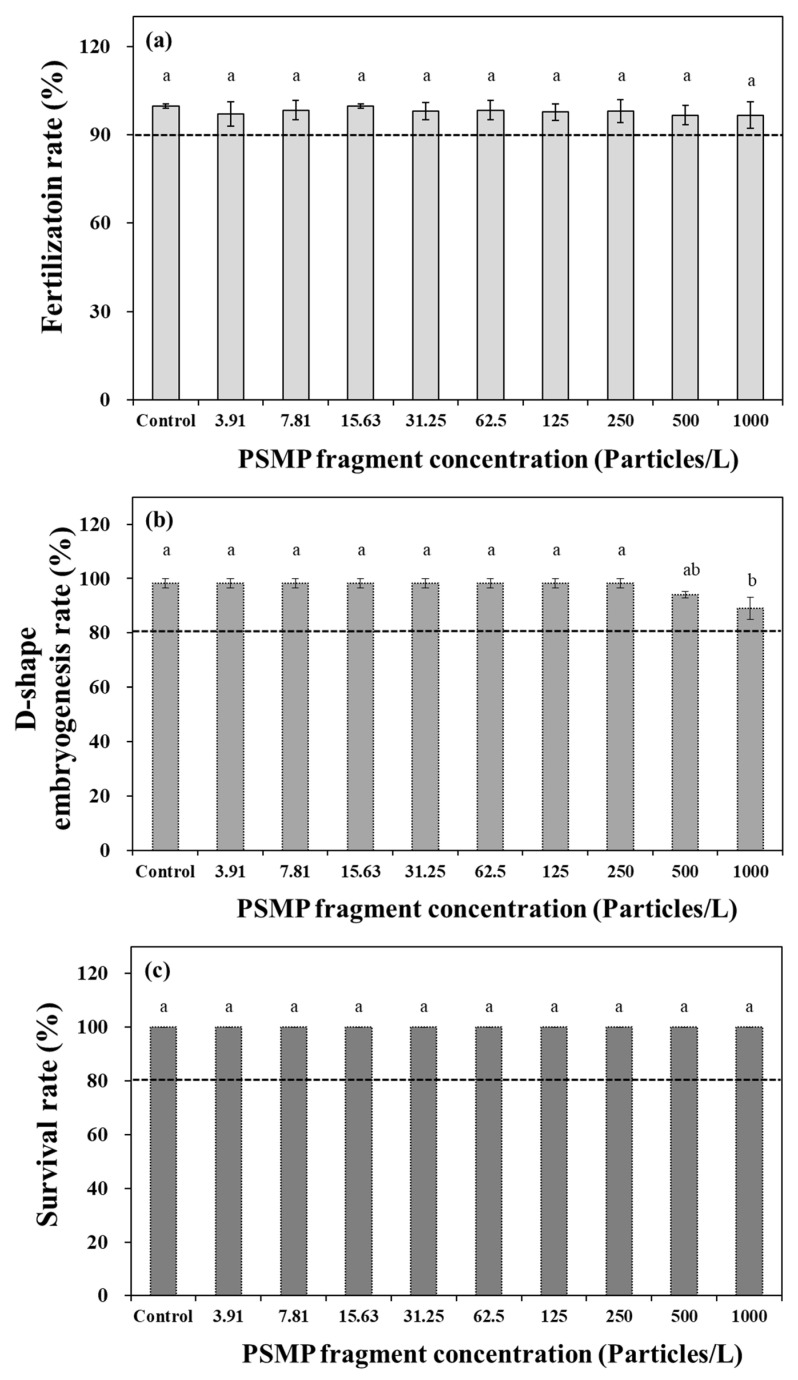
(**a**) Changes in the fertilization rate, (**b**) D-shaped embryogenesis rate, and (**c**) survival rate of *Crassostrea gigas* exposed to PSMPs. Mean values represent the responses to dose (*n* = 3), and the vertical bars denote ± 95% confidence intervals (*p* < 0.05). Values with different superscripts (a, b, and ab) were determined using Tukey’s multiple range test (*p* < 0.05) to indicate that they are significantly different from each other. PSMP, polystyrene microplastic particles.

In this study, we performed three ecological toxicity assessments on *C. gigas* using manufactured PSMP fragments (0–1000 particles/L). The fertilization rate of *C. gigas* was assessed based on the presence or absence of a fertilization membrane and polocyte following fertilization (Figure 1a,b). In addition, normal D-shaped embryonic development was evaluated by examining the morphological variations in embryo shape (Figure 1c,d). Abnormal embryonic development could result in morphological deformation. According to these bioassessments, the effect of PSMP exposure during *C. gigas* early developmental stages on the fertilization rate and D-shaped embryogenesis rate did not significantly differ from the acceptability criterion (Figure 2a,b). Similarly, in the mature stages, the survival rate of *C. gigas* remained unaffected by 1000 particles/L of PSMP fragments (Figure 2c). However, a slight but statistically significant inhibition (*p* < 0.05) of the D-shaped embryogenesis rate was observed at concentrations of >500 particles/L compared to the control group; however, it did not affect normal embryo development by more than 20%.

Our results, consistent with those of other studies (summarized in Table 2), suggest that MPs exclusively do not exhibit toxicity toward *C. gigas*. In addition to the bivalve used in this study, the endpoints of embryogenesis, photosynthesis, growth, blood circulation, and oxygen consumption in diatoms, microalgae, and fish remained unaffected by MP exposure. Furthermore, plain plastics such as PS, polyethylene, polyvinyl chloride, and polyethylene terephthalate revealed no toxicity toward various marine organisms compared to the control group (Table 2). In this study, *C. gigas* were simultaneously exposed to all micro sizes of MPs (1–1000 µm). Although this study reproduced the highest MP concentration reported in the surface seawater, toxicity was not observed at this concentration in the laboratory.

### 3.2. Combined Effect of Coastal Organic Pollutants with PSMPs

**Figure 3:**
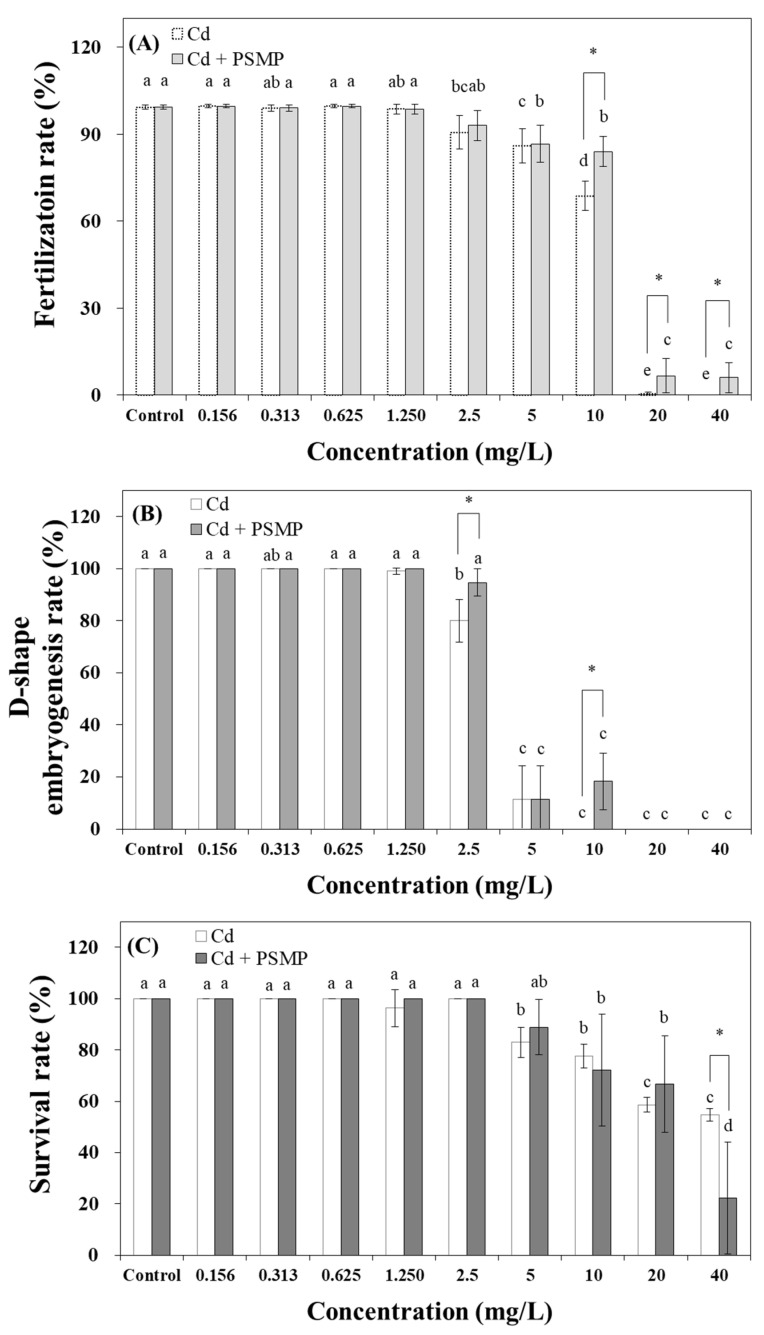
(**A**) Changes in the fertilization rate, (**B**) D-shaped embryogenesis rate, and (**C**) survival rate of *Crassostrea gigas* exposed to Cd in the presence or absence of PSMPs. Mean values represent the responses to dose (*n* = 3), and the vertical bars denote ± 95% confidence intervals (*p* < 0.05). Asterisks indicate a significant difference (*p* < 0.05 *) from the acceptability criterion determined using one-way ANOVA. Values with different superscripts (a, b, c, ab, bc, d, and e) were determined using Tukey’s multiple range test (*p* < 0.05) to indicate that they are significantly different from each other. PSMP, polystyrene microplastic particles.

**Figure 4:**
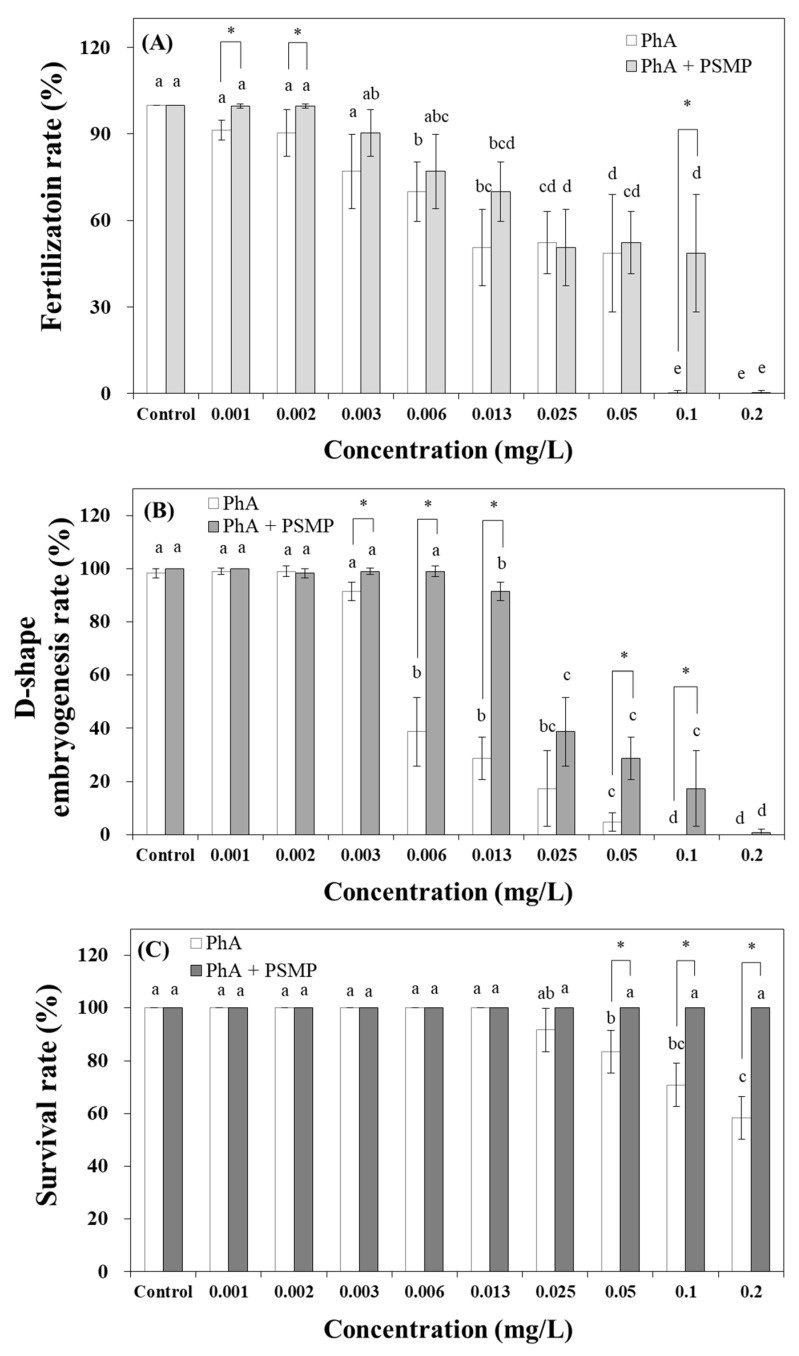
(**A**) Changes in the fertilization rate, (**B**) D-shaped embryogenesis rate, and (**C**) survival rate of *Crassostrea gigas* exposed to Phe in the presence or absence of PSMPs. Mean values represent the responses to dose (*n* = 3), and the vertical bars denote ± 95% confidence intervals (*p* < 0.05). Asterisks indicate significant differences (*p* < 0.05 *) from the acceptability criterion determined using one-way ANOVA. Values with different superscripts (a, b, c, d, e, ab, abc, bc, bcd, and cd) were determined using Tukey’s multiple range test (*p* < 0.05) to indicate that they are significantly different from each other. PSMP, polystyrene microplastic particles; Phe, phenanthrene.

In the pollutant toxicity test with Cd, the fertilization, D-shaped embryogenesis, and survival rates decreased significantly, at 2.5, 2.5, and 5 mg/L Cd concentrations, respectively, compared to the control (Figure 3). Similarly, the combined toxicity test with PSMPs using three endpoints decreased in a concentration-dependent manner. Meanwhile, the addition of PSMPs altered toxin susceptibility. Toxicity tests, such as the effects of PSMPs on fertilization and D-shaped embryogenesis rates, were performed during the early developmental stages of *C. gigas.* The addition of PSMPs led to a significant decrease (*p* < 0.05) in the fertilization rate at 10, 20, and 40 mg/L, compared to that observed with Cd exposure alone (Figure 3A). Similar changes were observed for the D-shaped embryogenesis rate at >2.5 mg/L PSMP addition (Figure 3B). However, in mature *C. gigas,* the toxic influence of Cd considerably increased. At 40 mg/L, the survival rate in the PSMP-supplemented sample was 22.2%, which was 32.4% lower than that observed in the Cd-only sample (Figure 3C). When *C. gigas* were exposed to Phe, the patterns observed in the three endpoints were identical to the Cd exposure results. The observed responses decreased as the concentration increased. However, the degree of toxic influence of PSMPs differed from that of Cd exposure alone. Simultaneous exposure to Phe and PSMPs visibly decreased the toxic effect of Phe. The fertilization rate was 0% at 0.1 mg/L Phe and increased to 48.7% after the addition of PSMPs. The Phe-only group differed from the Phe and PSMP groups at 0.001 mg/L. The Phe-exposed sample showed a 0% fertilization rate at 0.1 mg/L, whereas the samples containing Phe supplemented with MPs exhibited a 0% fertilization rate at 0.2 mg/L (Figure 4A). Similar patterns were observed for the D-shaped embryogenesis rate. In the Phe-only samples, the toxic effects were observed at 0.001 mg/L, whereas those of the Phe-MP samples began at 0.003 mg/L (Figure 4B). Phenanthrene toxicity remarkably affected the survival rate as the concentration increased; however, the toxic effect entirely ceased in the combined Phe-PSMP samples at 0.2 mg/L (Figure 4C). The effect of PSMPs on the two contaminants in *C. gigas* is summarized as follows: In general, the toxic effect of Phe and Cd on *C. gigas* decreased with PSMP addition; however, the toxicity increased in the mature stages of *C. gigas*. Thus, the effect of PSMPs may depend on the developmental stages of the organism and the PSMP concentration.

### 3.3. Toxicity Evaluation for the Interactions of MPs with Coastal Organic Pollutants

The toxicity response for Cd and Phe is represented by a sigmoid curve, which is considered a dose–response relationship (Figure 3 and Figure 4). The fertilization, D-shaped embryogenesis, and survival rates of *C. gigas* were evaluated using a toxic unit calculation to confirm the effect of PSMPs on the toxicity of Cd and Phe (Table 3). When *C. gigas* were exposed only to Cd, the EC_50_ values of the fertilization, D-shaped embryogenesis, and survival rates were 12.77 ± 0.26, 3.59 ± 0.13, and >40 mg/L, respectively, whereas, for the Cd-PSMP samples, the EC_50_ values were 14.43 ± 0.21, 3.90 ± 0.08, and 27.50 ± 1.19 mg/L, respectively. Moreover, when *C. gigas* were exposed to only Phe, the EC_50_ values of the fertilization, D-shaped embryogenesis, and survival rates were 15.25 ± 7.56, 7.96 ± 2.50, and 439.50 ± 175.43, respectively. However, for the combined Phe-PSMP samples, the EC_50_ values were 35.66 ± 1.27, 29.68 ± 15.96, and >200 mg/L, respectively (Table 3). The survival rate results were not sufficiently evident to perceive the change in toxicity. However, this tendency can be clearly observed from the evaluated toxic units. During Cd exposure, the addition of PSMPs increased the EC_50_ values (toxicity decreased) in the early life stages, and a decrease was observed during the adult life stages (toxicity increased). However, simultaneous exposure to Phe and PSMPs decreased the toxicity for all parameters, which is a prominent pattern in the survival rate. *Crassostrea gigas* mortality was not observed in the 0–0.2 mg/L Phe concentration range with PSMP addition. Therefore, the absorption of pollutants and discharge of MPs can alter the toxicity of pollutants, and the toxicity values, including NOEC, LOEC, EC_10_, and EC_50_, increased by 1.25–4.0 times (toxicity decreased) following PSMP addition.

Furthermore, PSMP is a hydrophobic substance with a high surface-to-volume ratio. Therefore, it can act as a carrier for hydrophobic chemicals. During transportation, the background pollutant level may be enhanced or reduced by continuous pollutant adsorption by MPs [8,89,90,91]. Therefore, simultaneous exposure of Cd and MPs indicates that adsorption by MPs can reduce the concentration of bioactive pollutants in the media/seawater/environment. As reported by Wang et al., MPs begin to rapidly adsorb Cd within 10 min of exposure, reaching saturation in approximately 90 min [92]. This adsorption ability of PSMPs can reduce the Cd concentration in seawater. Alternately, MPs can act as carriers by increasing toxicity through the “Trojan horse effect,” where the organisms adsorb Cd released from MPs [93]. Therefore, the Trojan horse effect may localize high concentrations of pollutants inside marine organisms [94,95]. Thus, simultaneous exposure to MPs in the adult stage (sufficient growth to absorb MPs) may increase toxicity because MPs act as a Cd carrier. However, research on microplastics as a medium for toxicity transfer via adsorption remains relatively scarce.

It is well established that environmental pollutants can adsorb onto microplastic surfaces through various interactions. First, “hydrophobic interactions” play a crucial role in the adsorption of hydrophobic plastics with nonpolar hazardous substances. Substances with high octanol–water partition coefficient (log Kow) values tend to be adsorbed more readily and in greater quantities [96,97,98,99,100,101]. Second, “electrostatic interactions” arise from attractive and repulsive forces due to charge differences between closely positioned molecules. Research has demonstrated that the polarity of microplastics varies with pH, facilitating adsorption through these electrostatic interactions [101,102,103,104]. Third, “hydrogen bonding” occurs when the hydrogen atom in a covalent bond interacts with an electronegative atom. This interaction commonly involves hydrogen covalently bonded to highly electronegative atoms such as oxygen, nitrogen, or fluorine, particularly in functional groups such as hydroxyl (-OH) and carboxyl (C(=O)OH) groups [101,105,106]. Fourth, van der Waals forces are weak physical interactions that allow microplastics to adsorb pollutants, even without specific functional groups on their surfaces [96,101,107,108]. Additionally, factors such as surface area, surface roughness, temperature, pH, and salinity may influence the interactions between microplastics and contaminants. In the context of this study, Cd can engage in electrostatic interactions and van der Waals forces; however, the impact of electrostatic interactions may be minimal due to the replicated experimental environment. Conversely, Phe can engage in hydrophobic interactions, hydrogen bonding, and van der Waals forces.

Several studies have carried out ecotoxicity tests using various factors governing MPs (size, material, and concentration) and coastal environmental pollutants. When marine organisms were simultaneously exposed to MPs and toxic substances, the toxicity increased, decreased, or remained unchanged. Moreover, the toxicity effects vary with species, pollutants, MP materials, and developmental stages (Table 4). For instance, when heavy metals, insecticides (chlorpyrifos), and pharmaceuticals (Sertraline) were exposed simultaneously with MPs (PE and PS), the toxicity increased [75,109,110]. Simultaneous exposure to PAHs (fluoranthene and Phe) and fungicides (triclosan) with MPs (polyethylene, polystyrene, and polyvinyl chloride) for various marine organisms, such as diatoms, bivalves, and copepods, decreased the toxicity of these pollutants [82,90,111,112]. However, several studies observed no changes, which cannot be easily explained, owing to differences in the types of substances (Table 4). For instance, PS reportedly exhibits the highest absorption force for POPs, compared with PE, PP, and PA [113]. Furthermore, Phe adsorption is higher (distribution coefficient, log Kd value is 3.07–4.20 L/kg) than that of other pollutants. Moreover, according to Wang et al., the Phe adsorption curve of PS is linear within 300 µg/L [114]. Therefore, the Trojan horse effect of Phe was not suspected in this study. Finally, the log Kow values of the Phe (4.46) and Cd (0.21) concentrations used in this study may be directly associated with the toxicity changes after MP addition. Thus, the background concentration, type of pollutants, and MPs in the environment simultaneously affect the toxicity levels.

Essentially, five factors may affect the increase or decrease in toxicity levels. The first factor is the difference in toxicity caused by sizes. In general, MP toxicity increases with a decrease in size [109,124]. Small-sized background MPs may exhibit self-toxicity, which may alter the result of combined toxicity. Second, the modes of action of toxicants differ by pollutant type, where each pollutant has a different mode of action leading to different toxicity levels. The third factor is the relationship between pollutant concentration and the log Kow. Hazardous and noxious substances with a high log Kow are quickly adsorbed to hydrophobic plastic polymers, leading to a lower, less toxic background concentration. However, the relevance may be diminished at concentrations that exceed the adsorption capacity. The fourth factor is the difference in the chemical adsorption ability of plastic materials. According to Xu et al. [113] the adsorption capacity of the material varies according to the plastic material in the following order: PS > PE > PP > PA [114]. The fifth factor is the variation in toxin sensitivity with the developmental stage of living organisms; moreover, as the developmental stages progress, the adsorption of larger-sized MPs increases, which can alter the tolerance to toxicity. Furthermore, it is dependent on pollutant exposure time. Therefore, to confirm the effects of the complex toxicity of MPs, the surface organisms’ life history, material characteristics, and the size and type of MP should be considered.

This study has two limitations. The first being the varying exposure times for PSMPs and pollutants, which depend on the test method used in toxicity tests with living organisms.

Additionally, the test method used in this study is an acute toxicity test; therefore, it is not representative of the entire life cycle or intergenerational toxicity. Moreover, the results may be underestimations due to differences in the evaluation methodologies caused by background concentration. Nevertheless, in this study, a life-stage-specific toxicity evaluation of *C. gigas* was performed, which is significant in that the toxic interaction of PSMPs with coastal environmental pollutants using bivalves was confirmed in conditions similar to those in their environment.

## 4. Conclusions

In this study, the toxic interactions of PSMPs with major organic pollutants, such as Cd and Phe, in coastal areas were investigated using three toxic endpoints, as follows: survival rates of *C. gigas* during the gamete stage (cellular level), early life stage (embryo level), and adult stage (individual level). The results showed that simultaneous exposure to PSMPs and pollutants reduced the toxicity of the pollutants. However, simultaneous exposure to Cd (40 mg/L) and PSMPs increased mortality in the adult stage, owing to the Trojan horse effect. As organic pollutants adsorb onto MPs (lowering the background concentration), their combination may alter pollutant toxicity. Therefore, the toxic interaction is influenced by the following criteria: the size of the MPs, the biotoxic mechanism of the pollutants, the concentrations and log Kow value of the chemicals, differences in MP adsorption ability, and the developmental stage and size of the target organism. Overall, the toxicity of MPs and their interaction with pollutants in the coastal environment are expected to affect their biotoxicity, which is a major environmental issue. This study systematically investigates potentially hazardous MPs and provides preliminary research into MP management strategies, indicating the importance of continuing MP research in the future.

## Figures and Tables

**Table 1:** Information on optimal experimental conditions. The fertilization and D-shaped embryogenesis rates were obtained using the standard method used for examining Korean marine environments. Survival rate analyses were performed in accordance with the USEPA method.

Class	Condition
Endpoint	1 h fertilization rate (%)
	30 h D-shaped embryogenesis rate (%)
	96 h survival rate (%)
Culture type	Static non-renewal
Photoperiod	Ambient light condition and 8L:16D period
Temperature	25 °C ± 0.5 °C
Salinity	32 ± 1.0
pH	8.0 ± 0.5
Test solution volume	10 mL (6-well plate) for early life stages
	2000 mL (beaker) for survival rate
Culture medium	Filtrated seawater (0.45 µm membrane filter)
Number of repeats	Three
Initial density of sperm cells and fertilized eggs	1 µL cleaned sperm/mL
	10–15 fertilized eggs/mL
Acceptability criterion	>90% fertilization rate
	>80% D-shaped embryogenesis rate
	>80% survival rate at control

**Table 2:** Summary of the effects of plain microplastics on marine species.

Common Name/ Species Name	Target Endpoint	MP Materials and Shape (Concentration)	MP Size	Observed Effect	Reference
Bivalve/ *Crassostrea gigas*	1 h fertilization rate, 30 h embryogenesis rate, 96 h survival rate	Polystyrene, Fragment (300 particles/L),	2–5000 µm	No effect on the fertilization and survival rates Inhibition less than 10%; could not meet the acceptance criteria	This study
Diatom/ *Phaeodactylum tricornutum*	72 h growth inhibition	Polyethylene, Bead (0–1.25 × 10^7^)	10–20 µm, 50–63 µm, 90–106 µm	Not significantly different from the control	[81]
Diatom/ *Phaeodactylum tricornutum*	96 h growth	Polyethylene, Bead (0–50 g/L),	150 µm	Not significantly different from the control; a slight difference was observed after 246 h of exposure	[82]
Microalgae/ *Isochrysis galbana*	72 h growth rate	Polyethylene, Bead (0.5–25 mg/L),	2–6 µm	Not significantly different from the control	[83]
Microalgae/ *Tetraselmis chuii*	96 h growth inhibition	Polyethylene, Bead (0–1.47 mg/L),	1–5 µm	Not significantly different from the control; however, 24% inhibition was observed	[84]
Microalgae/ *Tetraselmis chuii*	96 h growth inhibition, 96 h chlorophyll concentration	N.R, Bead (0–41.5 mg/L),	1–5 µm	Not significantly different from the control; however, concentration-independent changes were observed in chlorophyll concentration	[85]
Microalgae/ *Dunaliella tertiolecta*, *Thalassiosira pseudonana*, *Chlorella vulgaris*	72 h photosynthetic efficiency, 72 h growth inhibition	Polystyrene, Bead (25, 250 mg/L)	0.05–6 µm	No significant difference was observed in photosynthesis Growth was inhibited with exposure to small MP size (0.05 µm) with high density (250 mg/L); no change was observed for other concentrations and sizes on growth inhibition	[44]
Diatom/ *Skeletonema costatum*	96 h growth inhibition, 96 h photosynthetic efficiency, 96 h chlorophyll concentration	Polyvinyl Chloride, Bead (0–50 mg/L)	1 µm	Growth inhibition was concentration-dependent up to 50 mg/L for small sizes (1 µm). However, for the large size (1000 µm), no change was observed for concentrations of up to 2000 mg/L Chlorophyll concentration did not change, regardless of the size and concentration of PVC The amount of photosynthesis did not show any significant association with concentration or time	[86]
		Polyvinyl Chloride, Hexahedron (0–2000 mg/L)	1000 µm		
Fish/ *Danio rerio*	48 h velocity of blood flow, 48 h heartbeat	Polyethylene terephthalate, Bead (20 mg/L)	150 µm	No significant change regardless of the type of PET	[87]
		Polyethylene terephthalate, Fiber (20 mg/L)	0.3–0.5 mm (20 µm diameter)		
Fish/ *Danio rerio*	24 h mortality, 120 h hatching, 336 h growth, 456 h oxygen consumption	Polyethylene, Bead (0–20 mg/L)	1–45 µm	No significant difference was observed on mortality and hatching (for hatching, a transient decrease was observed at 48 h) No significant difference was observed in growth and oxygen consumption (for oxygen consumption, a transient decrease was observed at 168 h)	[88]

N.R: Not recorded.

**Table 3:** Toxicity evaluation using toxic parameters of *C. gigas* exposed to Cd and Phe in the presence or absence of PSMPs. Non-observed effective concentration (NOEC), lowest observed effective concentration (LOEC), and 50% and 10% effective concentration (EC_50_ and EC_10_, respectively) were calculated according to the guidelines provided by the USEPA using Toxcal 5.0 (Toxicalc 5.0, Tidepool Scientific Software, USA), Dunnett’s test, and likelihood probit analysis.

Toxicants	Toxic Parameter	Toxic Unit (Cd: mg/L; Phe: µg/L)			
		NOEC	LOEC	EC_10_	EC_50_
Cd	Fertilization rate	1.25	2.5	3.10 ± 0.95	12.77 ± 0.26
	D-shaped embryogenesis rate	1.25	2.5	1.84 ± 0.13	3.59 ± 0.14
	Survival rate	2.5	5	3.84 ± 0.29	>40
Cd + PSMPs	Fertilization rate	2.5	5	3.86 ± 1.19	14.43 ± 0.21
	D-shaped embryogenesis rate	2.5	5	2.65 ± 0.07	3.90 ± 0.08
	Survival rate	5	10	4.75 ± 1.19	27.50 ± 1.19
Phe	Fertilization rate	<0.78	0.78	1.52 ± 1.27	15.25 ± 7.56
	D-shaped embryogenesis rate	3.13	6.25	2.39 ± 1.33	7.96 ± 2.50
	Survival rate	12.50	25.00	48.01 ± 15.62	439.50 ± 175.43
Phe + PSMPs	Fertilization rate	1.56	3.13	3.87 ± 151.27	35.66 ± 1.27
	D-shaped embryogenesis rate	6.25	12.50	8.68 ± 2.94	29.68 ± 15.96
	Mortality rate	>200	>200	>200	>200

**Table 4:** Toxic interaction of PSMPs with pollutants. This table summarizes the increase or decrease in toxicity caused by MPs on the ecotoxicity of coastal environmental pollutants in marine organisms.

Toxicant Group	Target Toxicant (Concentration)	MP Materials and Type (Concentration)	MP Size	Common Name/ Species Name	Target Endpoint	Observed Effect	Reference
Heavy metal	Cadmium (0–40 mg/L)	Polystyrene (300 particles/L), Fragment	2–5000 µm	Bivalve/ *Crassostrea gigas*	1 h fertilization, 30 h embryogenesis, 96 h survival	Toxicity reduction in early life stages that increased in the adult stage	This study
	Cadmium (10 µg/L), Lead (50 µg/L), Zinc (100 µg/L)	Polystyrene, Bead (100 µg/L)	2.5 µm	Fish (marine medaka)/ *Oryzias melastigma*	30 d development, tissue pathology, microbiota	No significant changes were observed with development. Significant increase in accumulation when combined with MPs	[115]
	Copper (0.1–10 µg/L)	Polyethylene, Bead (100 mg/L)	4–6 µm, 11–13 µm, 20–25 µm	Bivalve/ *Crassostrea gigas*	24 h abnormality, development arrest, swimming speed	Toxicity induction observed when MPs exposed at the same time (small-sized MPs more effective)	[109]
PAHs	Phenanthrene (0–0.2 mg/L)	Polystyrene, Fragment (300 particles/L)	2–5000 µm	Bivalve/ *Crassostrea gigas*	1 h fertilization, 30 h embryogenesis, 96 h survival	Toxicity reduction (linear relationship between exposure time and toxicity reduction)	This study
	Phenanthrene (50 µg/L)	Polystyrene, Bead (0–200 µg/L)	10 µm	Fish (marine medaka)/ *Oryzias melastigma*	28 d growth, 3 d embryo malformation	Significantly decreased at 2 µg/L of MPs, but did not change when exposed to single Phe at 200 µg/L of MPs	[116]
	Phenanthrene (0.8 mg/L)	Polyethylene, Polyvinyl chloride, Bead (0–50 g/L)	150 µm, 250 µm	Diatom/ *Phaeodactylum tricornutum*	4–9 d growth	(Only MP) Non-toxic effect after 4 d, however, growth reduction after 9 days of exposure (Combined) toxicity reduction (decrement: PE > PVC)	[82]
	Fluoranthene (30 µg/L)	Polystyrene, Bead, (32 µg/L)	2, 6 µm	Bivalve/ *Mytilus edulis*, *Mytilus galloprovincialis*	7 d hematology, histopathological effect, oxidative stress	(Only MP) Hemocyte mortality and oxidative stress increased. Histopathological abnormality observed (Combined MP) Reduced toxicity in the short term, high accumulation of pollutants in the long term	[111]
	Fluoranthene (28, 84 µg/L), Phenanthrene (159, 415 µg/L)	Polystyrene: 109 mg/L, Polyethylene: 15–450 mg/L, Bead	PS: 10 µm PE: 3–221 µm	Copepod/ *Acartia tonsa, Calanus finmarchicu*	48–96 h mortality	Toxicity reduction observed	[90]
	Petroleum Hydrocarbons (50, 100 µg/L)	Polystyrene, Bead (0.26 mg/L)	30 µm	Bivalve/ *Tegillarca granosa*	14 d immunotoxicity, oxidative stress, genotoxicity	(Toxicity increase) Cell viability, DNA damage, oxidative stress, phagocytic activity, some gene expression (Toxicity reduction)	[117]
POPs	17β-estradiol, (0.1, 1 µg/L) benzo[α]pyrene (5, 50 µg/L)	Polystyrene, Bead (1 mg/L)	0.5, 30 µm	Bivalve/ *Tegillarca granosa*	4 d immunotoxicity, oxidative stress, genotoxicity	Large-sized MPs reduced the toxic effect and small-sized MPs induced a toxic effect	[118]
PBDEs	BDE-209 (10–100 µg/L)	Polystyrene (0.125 mg/L)	2 µm	Bivalve/ *Chlamys farreri*	15 d hemocytes parameters, DNA fragmentation	(Phagocytic analysis) High toxicity reduction effect at high toxicity concentration (DNA damage) Toxicity increased but was not significantly different	[119]
Organic pollutants	4-nonylphenol (0–120 µg/L)	Polyethylene, Bead (0, 1, 10 mg/L)	5.5 µm	Sea urchin/ *Paracentrotus lividus*	7 d population growth	Non-toxic changes observed in fed larvae. For starved larvae, observation of influences that were not dependent on variables	[120]
	Nonylphenol (0–3 mg/L)	PA, Polyethylene, Polystyrene (10, 30, 50, 70, 100 mg/L)	13–150 µm	Microalgae/ *Chlorella pyrenoidosa*	96 h growth, oxidative stress	Size (toxicity decreased up to 48 h, then increased) and material (PE ≈ PS > PA)-dependent toxicity decreased	[121]
	4-n-nonylphenol, (4.2–77 mg/L), 4-Methylbenzylidene-camphor (72–338 mg/L)	Polyethylene, Bead (1–10 mg/L)	4–6 µm	Copepoda/ *Acartia clause*, Sea urchin/ *Paracentrotus lividus*	48 h survival, development	No significant toxicity changes observed	[122]
	Tributyltin (0–50 µg/L)	Polystyrene, Bead (0, 1, 10 µg/L)	0.05 µm	Rotifer/ *Branchionus koreanus*	24 h mortality	(Toxicity reduction) LC_50_ values increased when MPs were added to the toxicant	[123]
Insecticide	Chlorpyrifos (0–100 µg/L)	Polyethylene, Fragment (0–100 µg/L)	1.4–42 µm (mean 7.73 µm)	Copepoda/ *Acartia tonsa*	24 h, 48 h, survival, feeding, egg production, hatching, recruitment	Toxicity was induced with simultaneous exposure to MPs	[75]
	Chlorpyrifos (0–3 µg/L)	Polyethylene, Bead (0.5–25 mg/L)	2–6 µm	Microalgae/ *Isochrysis galbana*	Growth rate	Toxicity increased when MPs were added at 2 mg/L concentration Toxicity decreased when MPs were added at 2–3 mg/L concentration	[83]
Pharmaceutical	Sertraline (0.1 µg/L)	Polystyrene, Bead, (0.29 mg/L)	0.5, 30 µm	Bivalve/ *Tegillarca granosa*	14 d hematology, ATP content, stress enzyme concentration	No effect of micro-sized MPs but synergistic toxic effect of nano-sized MPs	[110]
Bactericide, Fungicide	Triclosan (Single: 0–0.4 mg/L, Joint: 0.3 mg/L)	Polyethylene, Polystyrene, Polyvinyl Chloride (Single: 0–0.1 g/L) Joint: 50 mg/L)	1–74 µm	Diatom/ *Skeletonema costatum*	96 h growth, oxidative stress	Toxicity reduction (PE < PVC < PS < small-sized PVC)	[112]

## Data Availability

The datasets used and/or analyzed during the current study are available from the corresponding author upon reasonable request.

## Supplementary Materials

The following supporting information can be downloaded at: https://www.mdpi.com/article/10.3390/nano15050349/s1, Table S1: Size distribution of manufactured polystyrene microplastic fragments in seawater, measured before use in the *Crassostrea gigas* exposure experiment.

## Abbreviations

ANOVA	analysis of variance
BCF	bioconcentration factor
LD	linear dichroism
L_50_	50% effective concentration
LOEC	lowest observed effective concentration
MP	microplastic
PAH	polycyclic aromatic hydrocarbon
NOEC	non-observed effective concentration
Phe	phenanthrene
POP	persistent organic pollutant
PS	polystyrene
PSMP	polystyrene microplastic particles
