# Supplementary material for: Evaluating Toxic Interactions of Polystyrene Microplastics with Hazardous and Noxious Substances Using the Early Life Stages of the Marine Bivalve Crassostrea gigas
Source: Nanomaterials (Basel). 2025 Feb 24;15(5):349. doi: 10.3390/nano15050349 (PMC11901870; doi:10.3390/nano15050349)
Supplement: Supplementary file 1 [file nanomaterials-15-00349-s001.zip › nanomaterials-3440507-supplementary.pdf]

Supplementary Materials

Evaluating Toxic Interactions of Polystyrene Microplastics with Hazardous and Noxious Substances Using the Early Life Stages of the Marine Bivalve *Crassostrea gigas*

Hoon Choi [1,2,*,†], Un-Ki Hwang [2,†], Moonjin Lee [1], Youn-Jung Kim [3] and Taejun Han [4]

1 Maritime Safety and Environmental Research Division, Korea Research Institute of Ships and Ocean Engineering (KRISO), Daejeon 34103, Republic of Korea; moonjin.lee@kriso.re.kr
2 Tidal Flat Research Institute, Marine Environment Research Division, National Institute of Fisheries Science (NIFS), Busan 46083, Republic of Korea; vngi1@korea.kr
3 Department of Marine Science, Incheon National University, 119, Academy-ro, Yeonsu-gu, Incheon 22012, Republic of Korea; duckyj@incheon.ac.kr
4 Department of Animal Sciences and Aquatic Ecology, Ghent University, Westenschapspark 1, Bluebridge, 8400 Oostende, Belgium; taejun.han@ghent.ac.kr
* Correspondence: lv85choi@gmail.com; Tel.: +82-42-866-3363; Fax: +82-42-866-3624
† These authors contributed equally to this manuscript.

Table S1. Size distribution of manufactured polystyrene microplastic fragments in seawater, measured before use in the *Crassostrea gigas* exposure experiment.

	Size distribution (μm)							
	< 50	50-100	100-150	150-300	300-500	500-1000	1000 <	Total
Concentration of MPs (/L)	190.10	39.93	26.73	22.77	16.50	3.63	0.33	300.00
